# Modern Sociology

**Published:** 1897-02-06

**Authors:** 


### Modern Sociology.

It is a trite saying that the boy is father to the man. Yet in all our dealings with the criminal population, and in all our attempts not merely to punish crime but to diminish its prevalence, it is of the extremest importance to bear in mind that punitive and deterrent nfluences brought to bear upon the full-grown criminal can have but small influence on the sum total of crime so long as a yearly tale of juvenile offenders is being added to the roll.

In a very interesting book on the Juvenile Offender, Mr. Morrison* tells us that both in England and America an increase in the amount of juvenile crime s going on. Both juvenile crime and crime as a whole proceed in the main from the same conditions, one of the first and most conspicuous of which is the concentration of population arising out of the concentration of manufacturing industries. "All over the civilised world most crime is committed in districts which are most thickly populated." In these centres a decadent race springs up; the struggle for life is keener and more unscrupulous; cupidity is more highly and more frequently excited; a larger homeless population exists; men are more in the position of strangers to each other, and offences are less easily detected. Large cities then become the principal hotbeds of delinquency, and wherever there is a high rate of crime in general there a correspondingly high rate of juvenile crime is also found.

The strongest temptation of the ordinary juvenile, especially when hungry, is the impulse to steal, and this is stimulated in every street by interminable lines of shops exhibiting all kinds of merchandise in a half-protected state. But there are other causes, among which must be noted the disastrous influence which oscillations in the state of trade have upon the least fit members of town populations. Town life, far more than life in the country, tends to produce a large proportion of weak and ineffective people who are physically ill-fitted for fighting the battle of life. It is on these people that the oscillations of trade tell the first. The obtaining of work depends on two conditions—personal competence and the state of trade; and in times of depression it is the least competent who are first discharged, and it is from the ranks of these least fit people that, even among adults, the criminal classes are principally recruited. The physical and mental condition of our juvenile offenders becomes, then, a matter of very great interest, for it is largely owing to their unfitness for the ranks of labour that they drift into the ranks of crime.

The first thing that is observed in regard to this is the very high death-rate which obtains in our industrial schools. Mr. Morrison calculates that, including those discharged as hopelessly diseased, "sent away in a moribund condition," the death and moribund rate in boys' industrial schools amounts to an annual average of 9 per 1,000, whilst in girls' industrial schools the death and moribund rate amounts to 12 per 1,000, the death-rates for boys and girls of corresponding ages outside being 3·7 and 3·8 respectively. Nothing could more strikingly indicate the bodily decadence which prevails among juveniles addicted to crime than this enormous mortality.

An additional proof, however, of the hereditary influences which are in operation in regard to this class is derived from the proportion of orphans which occur among these children. Premature death, when not produced by violence, is a sure sign of feeble vitality, and when we find that thirty-nine in every hundred of the inmates of our industrial schools are orphans, on one side or both, we may be sure that these children are the offspring of a decadent stock, and that, as a result even of hereditary influence alone, large numbers of them are burdened with diseased and debilitated constitutions, such as greatly to unfit them for the struggle by which alone they can obtain proper living in ordinary industrial pursuits.

The way in which some children are handicapped in the race, and the degree in which such weaklings drift into the ranks of crime, are well shown by a comparison of the physical development of children in different classes of society in the British Isles, derived from some statistics collected by a committee of the British Association and published in 1883. Now, among the general population, boys in public schools are the tallest for their age; next in height are boys in middle-class schools; after them the children in elementary schools; and last of all the children in industrial schools. Between the children in public schools and those in industrial schools there is a difference of no less than five inches in height at corresponding ages.

It is then very obvious that the juvenile offenders who inhabit our industrial schools are not normal children, and are not the offspring of normal parents; but are children whose physical defects must have a detrimental influence on their industrial career. They earn their bread with greater difficulty than their compeers; they never become properly incorporated with the army of labour; they hang upon its skirts and pick up what the ordinary artisan has left behind; and at the first turn of trade are thrown on the streets, and have to choose between a life of pauperism and a life of crime. Physical deficiency and mental incompetence are then at the root of a very large proportion of juvenile crime, and much as we may attempt by punishment, by detention in prison, and by reformatory efforts to lessen the number of the criminal classes, we have to face the fact that by the conditions of life which we allow to exist in our great cities, and, by way of the overcrowding of these cities, which we actually encourage by the general drift of our fiscal legislation, we every year produce and pour upon the industrial market this great residuum of immature and imperfect weaklings.

Could we not do more than we do to prevent this constant manufacture of habitual criminals which has its root in juvenile crime? Not more than 15 per cent. of the juveniles committed to reformatory and industrial schools come from homes in which they are fairly housed, fairly fed, and fairly clad; while of the young people between sixteen and eighteen who are committed to prison, 46 per cent. have no settled home, and usually resort to common lodging-houses for food and shelter. The common lodging-house still remains in too many cases the starting-point of a criminal career. It may be contrary to economic laws for municipalities to undertake the vast work of providing homes for the working classes; but in view of the immense importance to the community of stopping this manufacture of criminals at its very source, it would seem well within the range of municipal duty to provide some form of decent shelter for these children—for they are but children—who drift into crime chiefly because they are thrown upon the world while weakly and immature.

* "Juvenile Offenders." By William Douglas Morrison. (London: T. Fisher Unwin. 1896.)

